# Molecular Evolution of Human Coronavirus 229E in Hong Kong and a Fatal COVID-19 Case Involving Coinfection with a Novel Human Coronavirus 229E Genogroup

**DOI:** 10.1128/mSphere.00819-20

**Published:** 2021-02-10

**Authors:** Susanna K. P. Lau, David C. Lung, Emily Y. M. Wong, Kam Leng Aw-Yong, Antonio C. P. Wong, Hayes K. H. Luk, Kenneth S. M. Li, Joshua Fung, Tony T. Y. Chan, James Y. M. Tang, Longchao Zhu, Cyril C. Y. Yip, Sally C. Y. Wong, Rodney A. Lee, Owen T. Y. Tsang, Kwok-Yung Yuen, Patrick C. Y. Woo

**Affiliations:** a Department of Microbiology, Li Ka Shing Faculty of Medicine, The University of Hong Kong, Hong Kong, China; b Carol Yu Centre for Infection, Li Ka Shing Faculty of Medicine, The University of Hong Kong, Hong Kong, China; c Department of Pathology, Queen Elizabeth Hospital, Hong Kong, China; d Department of Pathology, Pamela Youde Nethersole Eastern Hospital, Hong Kong, China; e Department of Medicine and Geriatrics, Princess Margaret Hospital, Hong Kong, China; Georgetown University

**Keywords:** COVID-19, HCoV-229E, SARS-CoV-2, human coronavirus

## Abstract

Compared to other human coronaviruses, the genetic diversity and evolution of human coronavirus 229E (HCoV-229E) are relatively understudied. We report a fatal case of COVID-19 pneumonia coinfected with HCoV-229E in Hong Kong. Genome sequencing of SARS-CoV-2 and HCoV-229E from a nasopharyngeal sample of the patient showed that the SARS-CoV-2 strain HK13 was most closely related to SARS-CoV-2 type strain Wuhan-Hu-1 (99.99% nucleotide identity), compatible with his recent history of travel to Wuhan. The HCoV-229E strain HK20-42 was most closely related to HCoV-229E strain SC0865 from the United States (99.86% nucleotide identity). To investigate if it may represent a newly emerged HCoV-229E genotype in Hong Kong, we retrieved 41 archived respiratory samples that tested positive for HCoV-229E from 2004 to 2019. Pneumonia and exacerbations of chronic airway diseases were common among infected patients. Complete RdRp, S, and N gene sequencing of the 41 HCoV-229E strains revealed that our contemporary HCoV-229E strains have undergone significant genetic drift with clustering of strains in chronological order. Two novel genogroups were identified, in addition to previously described genogroups 1 to 4, with recent circulating strains including strain HK20-42 belonging to novel genogroup 6. Positive selection was detected in the spike protein and receptor-binding domain, which may be important for viral evolution at the receptor-binding interphase. Molecular dating analysis showed that HCoV-229E shared the most recent common ancestor with bat and camel/alpaca 229E-related viruses at ∼1884, while camel/alpaca viruses had a relatively recent common ancestor at ∼1999. Further studies are required to ascertain the evolutionary origin and path of HCoV-229E.

**IMPORTANCE** Since its first appearance in the 1960s, the genetic diversity and evolution of human coronavirus 229E (HCoV-229E) have been relatively understudied. In this study, we report a fatal case of COVID-19 coinfected with HCoV-229E in Hong Kong. Genome sequencing revealed that our SARS-CoV-2 strain is highly identical to the SARS-CoV-2 strain from Wuhan, compatible with the patient’s recent travel history, whereas our HCoV-229E strain in this study is highly identical to a recent strain in the United States. We also retrieved 41 archived HCoV-229E strains from 2004 to 2019 in Hong Kong for sequence analysis. Pneumonia and exacerbations of chronic airway diseases were common diagnoses among the 41 patients. The results showed that HCoV-229E was evolving in chronological order. Two novel genogroups were identified in addition to the four preexisting HCoV-229E genogroups, with recent circulating strains belonging to novel genogroup 6. Molecular clock analysis dated bat-to-human and bat-to-camelid transmission to as early as 1884.

## INTRODUCTION

Human coronaviruses (CoVs) were historically considered of low virulence, with their clinical and public health impact largely ignored. CoVs are classified into four genera, *Alphacoronavirus*, *Betacoronavirus*, *Gammacoronavirus*, and *Deltacoronavirus*. Before the severe acute respiratory syndrome (SARS) epidemic in 2003, only two human CoVs, HCoV-229E (alphacoronavirus) and HCoV-OC43 (betacoronavirus), were recognized, which were mainly associated with common colds. Since the discovery of SARS-CoV (betacoronavirus) and its animal origins (1–7), numerous CoVs have been discovered, including two additional human coronaviruses, HCoV-NL63 (alphacoronavirus) and HCoV-HKU1 (betacoronavirus) (8–11). The emergence of Middle East respiratory syndrome CoV (MERS-CoV) in 2012 and SARS-CoV-2 recently (both betacoronaviruses) showed that CoVs are important human pathogens.

Compared to other human CoVs, relatively little is known about the genetic diversity and evolution of HCoV-229E. Since the first isolation of HCoV-229E in 1965 as a novel common cold virus from organ culture (12), only 24 complete genome sequences of HCoV-229E have been available in GenBank, including one from the cDNA clone Inf-1 of the laboratory-adapted strain VR-740 (13) and 23 from clinical isolates in the United States, Italy, Netherlands, Germany, and Haiti (14–16). In one early study, sequencing of the spike genes of three geographically and chronologically distinct HCoV-229E strains showed rather limited variations (17). However, a subsequent study demonstrated genetic drift in the spike and nucleoprotein genes between 25 chronologically distinct HCoV-229E strains circulating in Australia between 1979 and 2004, which were clustered into four groups, group 1 to 4 (18).

In this study, we report a fatal case of coronavirus disease 2019 (COVID-19) coinfected with HCoV-229E. To investigate if the HCoV-229E strain may represent a newly emerged genotype, we also retrieved archived respiratory samples that tested positive for HCoV-229E in Hong Kong and performed complete RdRp, S, and N gene sequencing. The clinical characteristics of patients were analyzed in relation to molecular epidemiology data. Molecular dating was performed to understand the evolution of HCoV-229E.

## RESULTS

### A fatal case of COVID-19 coinfected with HCoV-229E.

A 39-year-old Chinese man with a history of diabetes mellitus was admitted to hospital in late January 2020 with progressive shortness of breath 1 week after returning from Wuhan. On admission, his temperature was 38.2°C with 100% oxygen saturation at room air. Complete blood count showed normal leukocyte count but lymphopenia (0.9 × 10^9^/liter). High-resolution computed tomography of thorax showed bilateral multifocal ground-glass opacities, compatible with COVID-19. His nasopharyngeal aspirate (NPA) for SARS-CoV-2 quantitative reverse transcription PCR (qRT-PCR) and COVID-19 loop-mediated isothermal amplification (LAMP) (19) were positive. HCoV-229E was also detected by multiplex PCR for respiratory pathogens (BioFire FilmArray). He was initially treated with amoxicillin-clavulanate and doxycycline and later lopinavir/ritonavir and interferon beta-1b. Despite treatment, he developed hypothermia and cardiac arrest 4 days after admission and failed resuscitation.

### Complete genome sequencing of SARS-CoV-2 and HCoV-229E from the fatal COVID-19 case.

**FIG 1:**
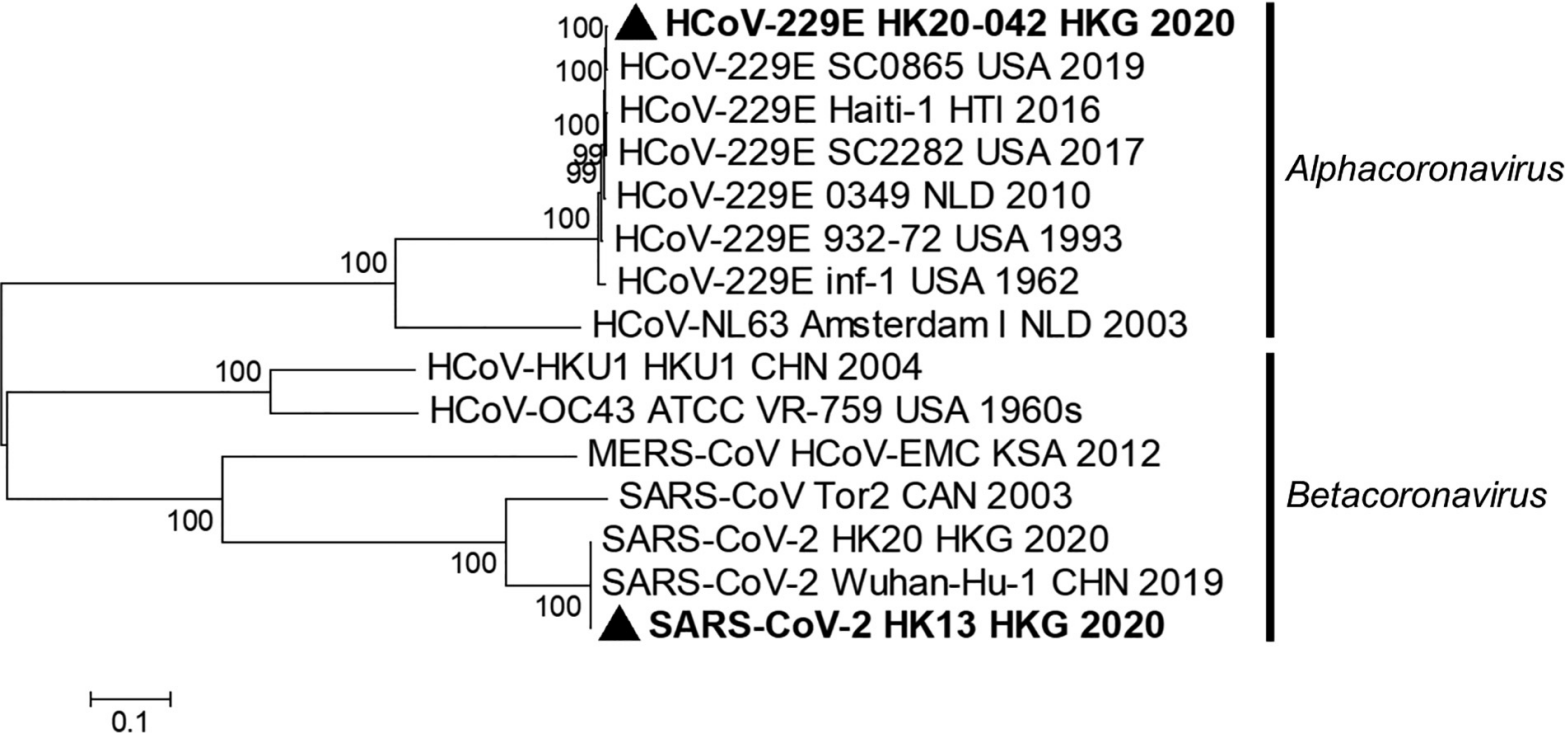
Phylogenetic analysis of complete genomes of the SARS-CoV-2 and HCoV-229E strains from the fatal case. The tree was constructed by maximum likelihood with GTR + G + I substitution model. Bootstrap values were calculated from 1,000 trees, and only bootstrap values over 70% are shown. Both viral strains of HCoV-229E and SARS-CoV-2 sequenced in this study are in bold font and marked with triangles. Country codes are as follows: CAN, Canada; CHN, China; HKG, Hong Kong; HTI, Haiti; KSA; Kingdom of Saudi Arabia; NLD, The Netherlands; USA, United States.

The genome sequence of SARS-CoV-2 strain HK13 from the patient (patient 42 in Table 1) shared 99.99% and 99.98% nucleotide identities to SARS-CoV-2 type strain Wuhan-Hu-1 (GenBank accession no. NC_045512) and another SARS-CoV-2 strain, HK20 (GenBank accession no. MT186683), previously detected in Hong Kong, respectively (20). The genome sequence of the HCoV-229E strain HK20-42 shared 97.89% nucleotide identities to that of HCoV-229E Inf-1 reference sequence (GenBank accession no. NC_002645) but was most closely related to that of HCoV-229E SC0865 (GenBank accession no. MN306046) with 99.86% nucleotide identities. The phylogenetic relationship of SARS-CoV-2 HK13 and HCoV-229E HK20-42 to other human CoVs is shown in Fig. 1.

**TABLE 1:** Clinical characteristics of the 42 cases with HCoV-229E infections in Hong Kong^*a*^

Strain no.	Mo/yr	Sex	Age	Underlying disease	Diagnosis	Group	CXR findings
HK04-01	Apr/2004	F	3 yr	None	Pneumonia	4	RLZ haziness
HK04-02	Jun/2004	M	14 yr	Thalassemia trait	Acute pharyngitis	4	Clear
HK05-03	Feb/2005	F	75 yr	DM, HT, appendicectomy	Pneumonia	4	RUZ consolidation, LUZ infiltrates
HK05-04	Mar/2005	F	2 yr	Biliary atresia, post-liver transplant	URTI	4	NA
HK05-05	Apr/2005	M	82 yr	Gallstones, cholecystectomy	URTI	4	Clear
HK05-06	Oct/2005	M	6 yr	NNJ, eosinophilic gastroenteritis, cleft lip and palate, phimosis, allergic rhinitis	Gastroenteritis	5	NA
HK05-07	Nov/2005	M	2 mo	NNJ	Pneumonia	5	RLZ haziness
HK05-08	Nov/2005	M	78 yr	COPD	COPD exacerbation	5	LMZ, RMZ, RUZ fibrosis
HK05-09	Nov/2005	F	33 yr	Depression	URTI	5	NA
HK05-10	Nov/2005	F	24 yr	Mental retardation, ASD, PDA with repair, CML, scoliosis with spinal fusion, granulomatous lymphadenitis	URTI	5	Clear
HK05-11	Dec/2005	M	57 yr	DM, HT, ESRF	Pneumonia	5	RLZ consolidation, blunted left CPA
HK05-12	Dec/2005	M	1 mo	None	URTI	5	NA
HK05-13	Dec/2005	F	79 yr	HT, CVA, hyperlipidemia, IHD, COPD	COPD exacerbation	5	Hyperinflated, prominent pulmonary trunk
HK05-14	Dec/2005	M	79 yr	Multiple myeloma, otitis media	Pneumonia	5	LLZ infiltrates
HK05-15	Dec/2005	F	24 yr	Catatonic schizophrenia, asthma	URTI, asthma	5	Clear
HK05-16	Dec/2005	F	82 yr	DM, HT, IHD, SSS, CHF, ESRF	URTI	5	Pulmonary congestion, pleural effusions
HK05-17	Dec/2005	F	7 yr	Short gut syndrome on TPN	URTI	5	Clear
HK05-18	Dec/2005	M	72 yr	COPD, BPH, allergic rhinitis	Pneumonia, COPD exacerbation	5	Apical fibrosis, loculated pneumothorax, RMZ haziness
HK05-19	Dec/2005	M	1 mo	None	URTI	5	Clear
HK05-20	Dec/2005	F	31 yr	None	URTI	5	Clear
HK05-21	Dec/2005	F	56 yr	ESRF, DU, parathyroidectomy, PTB	Pneumonia	5	Basal consolidation, apical thickening
HK05-22	Dec/2005	M	8 yr	None	Scarlet fever	5	Clear
HK06-23	Jan/2006	F	83 yr	Lymphoma, epilepsy	URTI	5	Clear
HK06-24	Jan/2006	F	96 yr	Dementia, HT, IHD, CVA, right NOF	Pneumonia, UTI	5	RLZ infiltrates
HK06-25	Jan/2006	M	19 mo	Asthma	URTI, asthma	5	Clear
HK06-26	Jan/2006	M	39 yr	None	Pneumonia	5	LLZ consolidation and left pleural effusion
HK06-27	Jan/2006	F	87 yr	CVA, epilepsy, recurrent pneumonia, bronchiectasis	Pneumonia	5	Left pleural effusion, LMZ airspace shadows
HK06-28	Jan/2006	F	95 yr	Metastatic CA lung, deafness	Pneumonia	5	RLZ and LLZ infiltrates, LMZ and LLZ nodules, RUZ fibrosis, pleural effusions
HK06-29	Jan/2006	F	65 yr	DM, schizoaffective disorder	URTI	5	Clear
HK09-30	May/2009	F	43 yr	DM, schizophrenia, pituitary microadenoma	Sinusitis	5	Clear
HK09-31	May/2009	M	1 yr	Laryngomalacia, developmental delay, recurrent bronchiolitis	Bronchiolitis	5	NA
HK11-32	Feb/2011	F	8 yr	None	URTI, tonsillitis	5	NA
HK11-33	May/2011	M	4 yr	AML relapse	URTI	6	NA
HK11-34	May/2011	F	5 mo	None	URTI, febrile convulsion	5	Clear
HK11-35	Jul/2011	M	90 yr	HT, renal failure, gout, dementia, aspiration pneumonia	Pneumonia	5	LMZ, LLZ haziness
HK12-36	Jan/2012	F	85 yr	HT, multiple myeloma, RPC	Pneumonia	6	RLZ consolidation
HK13-37	Feb/2013	F	70 yr	DM, HT	Pneumonia, UTI	6	RMZ infiltrate
HK13-38	May/2013	M	90 yr	HT, RAS, CVA, PVD, AAA, IHD, CHF, TB jaw	Pneumonia	6	RMZ, LLZ infiltrates
HK13-39	May/2013	M	2 yr	Nephrotic syndrome, asthma	Pneumonia, asthma	6	Perihilar haziness
HK16-40	Jan/2016	F	50 yr	SLE, miliary TB, splenic vein occlusion	Neutropenic fever	6	Clear
HK16-41	Jan/2016	M	62 yr	COPD, DU, IHD, epilepsy	COPD exacerbation	6	Clear
HK20-42	Jan/2020	M	39 yr	DM	Pneumonia	6	Bilateral ground glass infiltrates

a Abbreviations: AAA, abdominal aortic aneurysm; AML, acute myeloid leukemia; ASD, atrial septal defect; BPH, benign prostatic hyperplasia; CA lung, carcinoma of lung; CHF, congestive heart failure; CML, chronic myeloid leukemia; COPD, chronic obstructive pulmonary disease; CPA, costophrenic angle; CVA, cerebrovascular accident; CXR, chest X ray; DM, diabetes mellitus; DU, duodenal ulcer; ESRF, end-stage renal failure; HT, hypertension; IHD, ischemic heart disease; LLZ, left lower zone; LMZ, left middle zone; LUZ, left upper zone; NA, not available; NNJ, neonatal jaundice; NOF, fractured neck of femur; PDA, patent ductus arteriosus; PTB, pulmonary tuberculosis; PVD, peripheral vascular disease; RAS, renal artery stenosis; RLZ, right lower zone; RMZ, right middle zone; RPC, recurrent pyogenic cholangitis; RUZ, right upper zone; SLE, systemic lupus erythematosus; SSS, sick sinus syndrome; TB, tuberculosis; TPN, total parenteral nutrition; URTI, upper respiratory tract infection; UTI, urinary tract infection.

### Clinical characteristics of patients with HCoV-229E infections in Hong Kong.

To investigate the genetic relatedness of HCoV-229E strain HK20-42 with circulating HCoV-229E strains in Hong Kong, we retrieved archived nasopharyngeal samples from two regional hospitals that tested positive for HCoV-229E by RT-PCR from 2004 to 2019. A total of 41 HCoV-229E-positive samples were identified, and the clinical characteristics of the 41 patients are summarized in Table 1. Nineteen were males, and 22 were females. Twenty-six were adults, and 15 were children (median age 43 years, range from 1 month to 96 years). Notably, most patients were at the extremes of age (14 patients ≤8 years old and 15 patients ≥70 years old, among which four patients were ≥90 years old).

Upper respiratory tract infection (URTI) and pneumonia were the most common diagnoses. Exacerbations of asthma (patients 15, 25, and 39) or chronic obstructive pulmonary disease (COPD; patients 8, 13, 18, and 41) were also common. Sixteen patients (38%) presented with symptoms of URTI, with two patients complicated by asthmatic exacerbation, one by febrile convulsion, and one by tonsillitis. Of the other 16 patients with pneumonia, 10 were elderly, ≥70 years old. Yet, the 39-year-old male patient (patient 26) did not have underlying disease and presented with fever and cough after recent travel to mainland China. His NPA was also positive for influenza A virus. The 57-year-old male patient (patient 11) with pneumonia had also just returned from a trip to mainland China. The pneumonia was complicated by exacerbation of asthma (patient 39) or COPD (patient 18) in two patients.

Four other patients also had recent travel history before symptom onset, with three (patients 3, 19, and 39) having returned from mainland China and one (patient 20) from Indonesia. The father and grandmother of the 1-month-old neonate (patient 19) were noted to have recent respiratory illnesses. Four other patients (patients 2, 7, 32, and 34) also had a history of recent contact with household members or friends with respiratory illnesses. The 14-year-old boy (patient 2) reported members of his dragon boat racing team having similar respiratory symptoms.

Besides patient 26 with influenza A virus coinfection, three other patients (patients 13, 21, and 31) had coinfection by Haemophilus influenzae, Streptococcus pneumoniae, and Pseudomonas aeruginosa, respectively. Except for the 90-year-old man with pneumonia who died of secondary bacterial pneumonia caused by Klebsiella pneumoniae 1 month later, all 40 other patients survived.

### Phylogenetic analysis of complete RdRp, S, and N genes of HCoV-229E strains in Hong Kong.

**FIG 2:**
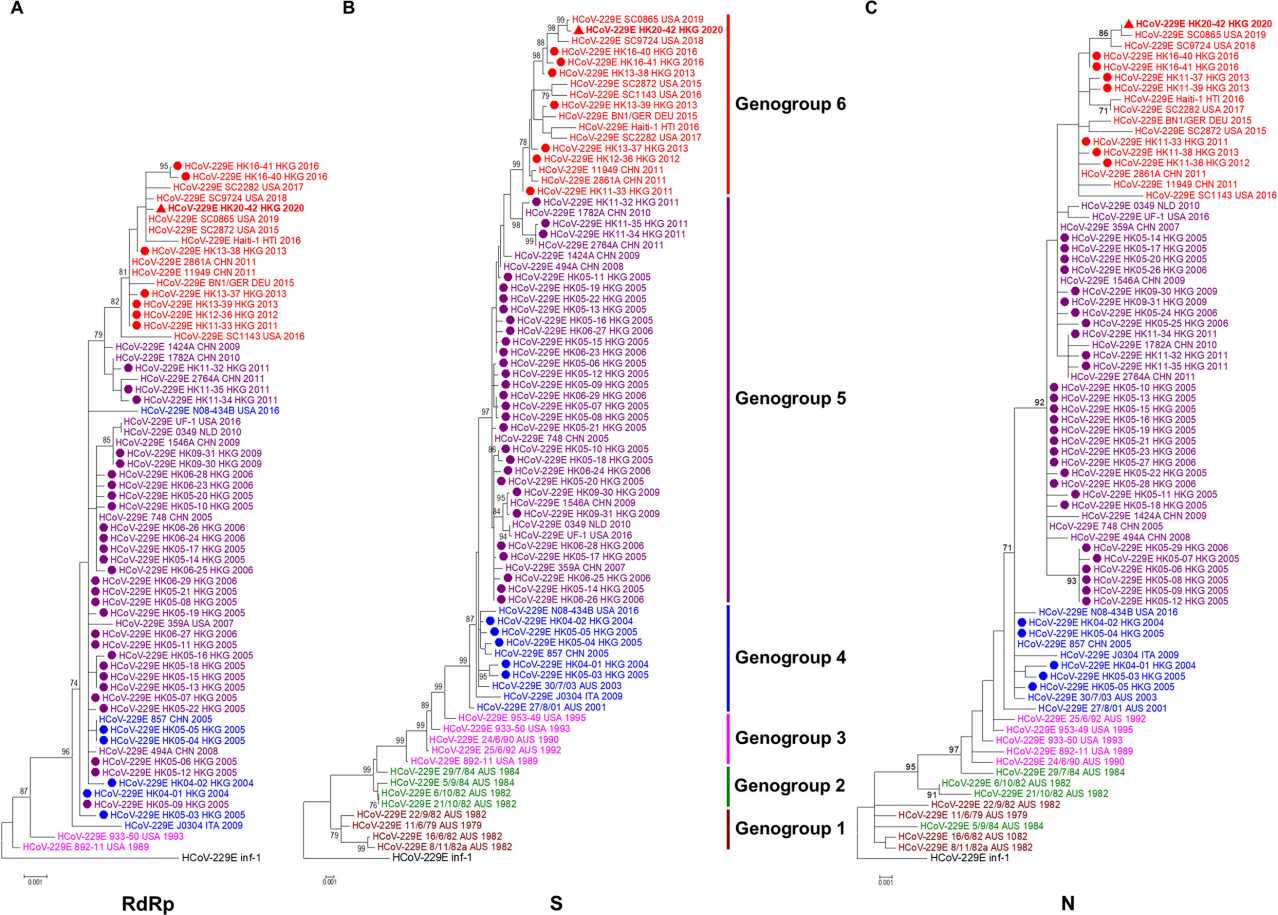
Phylogenetic analysis of the complete RdRp (A), S (B), and N (C) genes of HCoV-229E strains sequenced in this study. Trees were constructed using the maximum likelihood method by MEGA 7.0. Bootstrap values were calculated from 1,000 trees, and only bootstrap values over 70% are shown. The HCoV-229E strain from the COVID-19 fatal case is in bold font and marked with a triangle, and other HCoV-229E strains sequenced in this study are marked with circles. In the phylogenetic trees, different genogroups were indicated by different colors: black, HCoV-229E Inf-1 reference sequence; maroon, genogroup 1; green, genogroup 2; fuchsia, genogroup 3; blue, genogroup 4; purple, genogroup 5; red, genogroup 6. Country codes are as follows: AUS, Australia; CHN, China; DEU, Germany; HKG, Hong Kong; HTI, Haiti; ITA, Italy; NLD, The Netherlands; USA, United States.

To study the evolutionary relationship of the HCoV-229E strain HK20-42 with other local strains, the complete RdRp, S, and N genes of HCoV-229E from the 41 NPAs were amplified and sequenced. Strain HK20-42 and the other 41 HCoV-229E strains possessed 98.71 to 98.92%, 96.05 to 96.62%, and 97.52 to 98.21% nucleotide identities to the RdRp, S, and N sequence of HCoV-229E Inf-1 reference sequence, respectively. Phylogenetic analysis of the RdRp sequences showed that older HK strains from 2004 to 2006 (*n* = 29) and five HK strains from 2009 to 2011 were closely related, while eight HK strains from 2011 to 2020, including HK20-42, formed a distinct cluster with a high bootstrap value of 82% (Fig. 2A).

Phylogenetic analysis of S sequences demonstrated six distinct genogroups with different circulating periods. In line with a previous study (18), the older Australian strains formed three distinct clusters, with strains from 1979 to 1982 belonging to genogroup 1 and strains from 1982 to 1984 belonging to genogroup 2. Australian strains from 1990 to 1992 were closely related with United States strains in 1989 and 1995 to form genogroup 3. Genogroup 4 viruses were circulating during the period of 2001 to 2005, with the exception of Italy strain J0304 in 2009 and United States strain N08-434B in 2016. Five HK strains from 2004 to 2005 were closely related to a China strain from 2005 and Australian strains from 2001 to 2003 belonging to the previously described genogroup 4 (18), with nucleotide identities of 99.40% to 99.89%. The other 37 HK strains were only distantly related to strains belonging to genogroups 1 to 4. Together with strains detected from China from 2005 to 2011, Netherlands strain 0349 in 2010, and United States strain UF-1 in 2016, respectively, 29 HK strains from 2005 to 2011 fell into a new cluster (genogroup 5, circulating in period from 2005 to 2011), with a 97% bootstrap value. And together with strains from China detected in 2011, a Germany strain in 2015, a Haiti strain in 2016, and strains from the United States from 2015 to 2019, eight HK strains from 2011 to 2020, including HK20-42, formed another new cluster (genogroup 6), with a bootstrap value of 99% (Fig. 2B). The human aminopeptidase N (hAPN) is known to be the receptor for S protein binding in HCoV-229E, with the receptor-binding domain (RBD) located at amino acid positions 293 to 435 of the S1 region as determined by crystal structure analysis (21). The predicted RBD region of the 42 HK strains shared 89.04% to 90.44% nucleotide identity (78.32% to 79.72% amino acid identity) to the HCoV-229E Inf-1 reference sequence.

Phylogenetic analysis of N sequences showed tree topology similar to that of S genes, with the older Australian strains forming distinct clusters, though with some strains exhibiting slightly different phylogenetic positions. Five HK strains from year 2004 to 2005 were closely related to Australian genogroup 4 strains from 2001 to 2003, with nucleotide identities of 99.32% to 100%. Twenty-nine HK strains were distantly related to strains from genogroups 1 to 4 and formed a distinct cluster with a bootstrap value of 92%. Eight other HK strains from 2011 to 2020, including HK20-42, were closely related to each other to form another new cluster (genogroup 6) (Fig. 2C).

### Selective pressure analysis.

Using the 42 RdRp, S, and N gene sequences, the ratios of nonsynonymous substitutions per nonsynonymous site to synonymous substitutions per synonymous site (*K_a_*/*K_s_*) were calculated (Table 2). The highest *K_a_*/*K_s_* ratios were observed in the S gene (0.412), while RdRp (0.111) and N (0.143) genes showed lower *K_a_*/*K_s_* ratios. When the S gene of different genogroups was analyzed, the highest *K_a_*/*K_s_* ratio was observed in genogroup 6 (0.933), while the other genogroups showed much lower *K_a_*/*K_s_* ratios. *K_a_*/*K_s_* ratios (ω) in the S gene were calculated on a codon-by-codon basis, with most codons having ω of <1, indicating purifying selection (Table 2). Nevertheless, three codons were predicted to have a ω of >1 by at least two different methods with statistical significance, including codons 26 and 288 by fixed-effects likelihood (FEL) and random-effects likelihood (REL) methods, and codon 314 by REL and mixed-effects models of evolution (MEME) methods, indicating possible functional constraints at these positions. Codon 288 was predicted as a positively selected site among genogroup 6 by at least two methods, which accounts for the relatively high *K_a_*/*K_s_* ratio in genogroup 6. While residue 288 (Val) is conserved among the prototype and genogroups 1 to 5, V288A, V288M, and V288E were observed in genogroup 6. All three codons were distributed within the S1 domain, and codon 314 was situated within the RBD, indicating strong selective pressure at the receptor-binding interphase during viral evolution.

**TABLE 2:** Estimation of nonsynonymous and synonymous substitution rates in the RdRp, N, S, and receptor-binding domain of HCoV-229E strains^*a*^

Gene	*K_a_*	*K_s_*	*K_a_*/*K_s_*	Positive selected sites by MEME method	Positive selected sites by SLAC method	Positive selected sites by FEL method	Positive selected sites by REL method	Best fit model^*b*^
RdRp	0.001	0.009	0.111	Nil	Nil	Nil		001120
N	0.003	0.021	0.143	Nil	Nil	Nil		001120
S	0.007	0.017	0.412	125, 314		26, 288	22, 26, 90, 104, 213, 288, 305, 307, 311, 314, 316, 323, 325, 352, 357, 358, 407, 971, 1103	012030
Genogroup 1	0.002	0.024	0.083	Nil	Nil	Nil		010011
Genogroup 2	0.002	0.000		Nil	Nil	Nil	22, 125, 180, 350, 937, 1063	F81
Genogroup 3	0.007	0.000		Nil	Nil	Nil		HKY85
Genogroup 4	0.001	0.010	0.100	Nil	Nil	Nil	104	000010
Genogroup 5	0.002	0.011	0.182	Nil	Nil	Nil	89, 104, 175, 283, 325, 357	001120
Genogroup 6	0.014	0.015	0.933	Nil	Nil	288	90, 213, 288, 305, 311, 323, 358, 407, 739	HKY85

a Abbreviations: SLAC, single-likelihood ancestor counting; FEL, fixed-effects likelihood; MEME, mixed-effects model of evolution; REL, random-effects likelihood.

b Best fit model applied only for REL method; nucleotide GTR model was applied for SLAC, FEL, and MEME methods by default.

### Estimation of divergence time.

**FIG 3:**
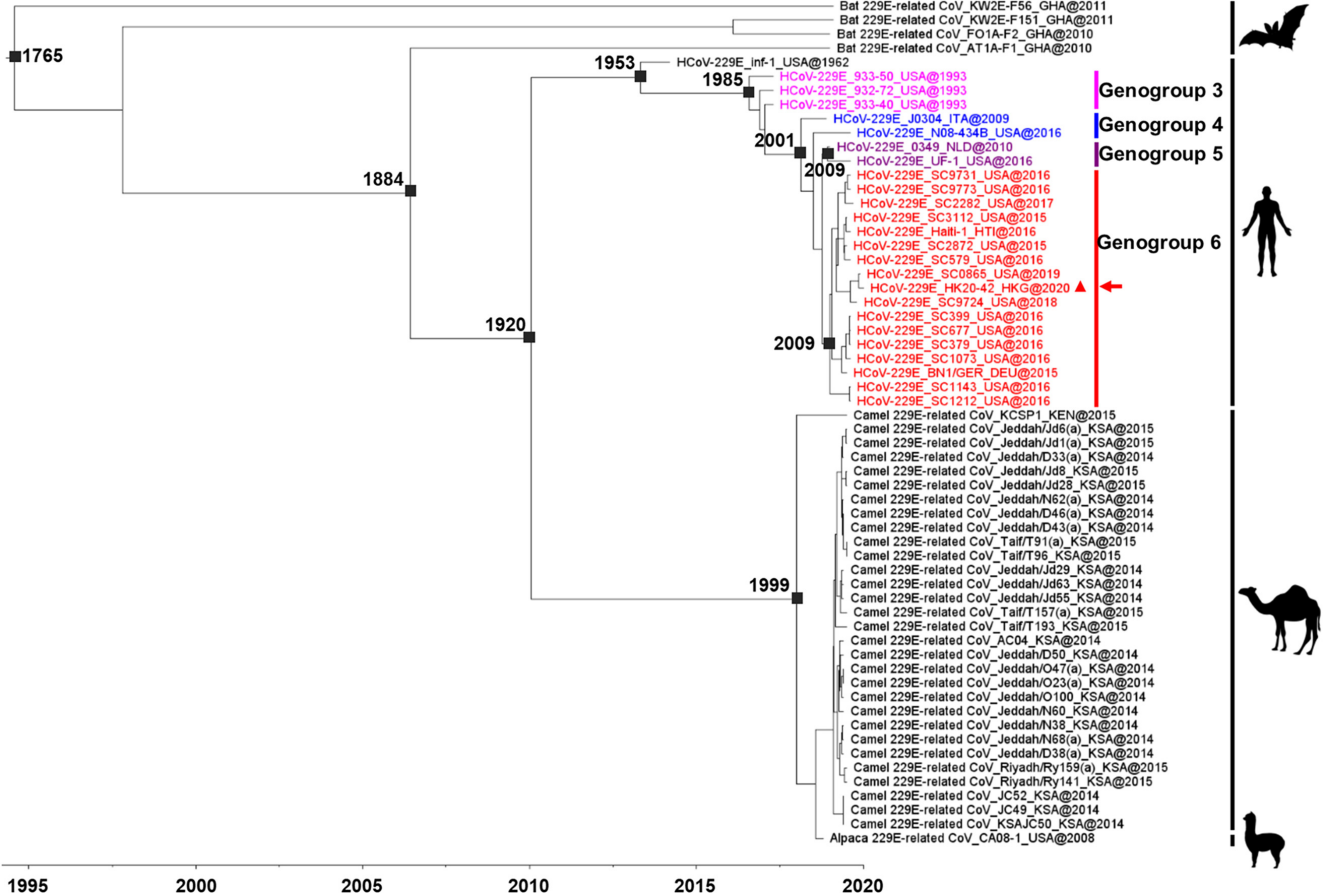
Estimation of the tMRCA of bat 229E-related CoVs, camel 229E-related CoVs, alpaca 229E-related CoVs, and HCoV-229E. The time-scaled phylogeny was summarized from MCMC phylogeny of the complete ORF1ab sequence data analyzed using the uncorrelated exponential relaxed clock and exponential growth coalescent model with an exponential distribution in BEAST v 1.8.2. The HCoV-229E strain from the COVID-19 fatal case is marked with a triangle and an arrow. The different genogroups of HCoV-229E are indicated by different colors: fuchsia, HCoV-229E genogroup 3; blue, HCoV-229E genogroup 4; purple, HCoV-229E genogroup 5; red, HCoV-229E genogroup 6. Country codes are as follows: DEU, Germany; GHA, Ghana; HKG, Hong Kong; HTI, Haiti; KEN, Kenya; KSA, Kingdom of Saudi Arabia; ITA, Italy; NLD, The Netherlands; USA, United States.

To understand the evolution of HCoV-229E and its divergence time from 229E-related CoVs in animals, their complete ORF1ab sequences were subjected to molecular clock analysis using the Bayesian uncorrelated exponential relaxed molecular clock and exponential growth coalescent model (Fig. 3). The estimated mean substitution rate was 3.03 × 10^−4^ (1.9 × 10^−4^ to 4.3 × 10^−4^) substitutions per site per year. The time of the most recent common ancestor (tMRCA) of all 229E-related CoVs was dated back to 1765 (95% highest-posterior-density region [HPD], 1722 to 1962), while that of bat 229E-related CoV AT1A-F1, camel/alpaca 229E-related CoVs, and HCoV-229E was estimated at 1884 (95% HPD, 1884 to 1885), suggesting that 229E-related CoVs may have jumped from bats to humans and other animals around 130 years ago. Interestingly, the tMRCA of HCoV-229E and camel/alpaca 229E-related CoVs was estimated at 1920 (95% HPD, 1915 to 1926), while that of HCoV-229E was estimated at 1953 (95% HPD, 1949 to 1958) and that of camel/alpaca 229E-related CoVs at 1999 (95% HPD, 2000 to 2000). This suggests that existing camel/alpaca 229E-related CoVs may have evolved from a relatively recent common ancestor, while HCoV-229E may have emerged in humans from other camels/alpacas or yet-unidentified animals between 1920 and 1953. For HCoV-229E, the tMRCA of genogroup 3 was estimated at 1985 (95% HPD, 1985 to 1987), genogroup 4 at 2001 (95% HPD, 2001 to 2002), genogroup 5 at 2009 (95% HPD, 2009 to 2010), and genogroup 6 at 2009 (95% HPD, 2010 to 2010). HCoV-229E genogroup 1 and genogroup 2 viruses were not included in the Bayesian analysis because no complete genome sequence was available.

## DISCUSSION

We report a fatal case of COVID-19 coinfected with HCoV-229E, the latter belonging to a new genogroup 6 arisen as early as 2011. The genome of the SARS-CoV-2 strain was highly similar to the type strain Wuhan-Hu-1, supporting the idea that the patient had acquired the infection from Wuhan before returning to Hong Kong. On the other hand, the HCoV-229E strain HK20-42 was genetically most closely related to HCoV-229E SC0865 detected in the United States in 2019. While no specific genome features are noted in HCoV-229E strain HK20-42 compared to other recently circulating strains of genogroup 6, it remains to be determined if coinfection with HCoV-229E may have contributed to the disease severity in the present case. Moreover, up to 20.7% of coinfections by SARS-CoV-2 and other respiratory pathogens were reported in the United States (22), suggesting that routine testing for other respiratory pathogens during the COVID-19 pandemic is unreliable to rule out SARS-CoV-2 infection.

The present study described the molecular diversity and evolutionary dynamics of HCoV-229E in Hong Kong. Although HCoV-229E has been known for more than half a century (12), evolutionary studies of HCoV-229E have been scarce. In this study, we showed that the contemporary HCoV-229E strains from Hong Kong have undergone significant genetic drift. Phylogenetic analysis of the S gene showed that HCoV-229E has continued to evolve with time to generate new genogroups, with clustering of strains in chronological order. The earliest group, genogroup 1, comprises strains detected from 1979 to 1982, followed by genogroup 2 comprising strains detected from 1982 to 1984, genogroup 3 comprising strains detected from 1989 to 1995, genogroup 4 comprising strains detected from 2001 to 2005, genogroup 5 comprising strains detected from 2005 to 2011, and a new genogroup 6, comprising strains detected from 2011 to 2020. The tMRCA of HCoV-229E was comparable with a previous report (23). The estimated mutation rate (substitutions per site per year) of HCoV-229E in the present study is similar to other previously reported rates of HCoV-229E (3.28 × 10^−4^ to 3.9 × 10^−4^) (23, 24), HCoV-NL63 (4.3 × 10^−4^) (23), HCoV-OC43 (3.6 × 10^−4^ to 8.48 × 10^−4^) (25–27), MERS-CoV (1.12 × 10^−3^) (28), SARS-CoV (0.8 × 10^−3^ to 2.38 × 10^−3^) (29), and SARS-CoV-2 (9.9 × 10^−4^) (30). The mutation rates of HCoVs are lower than some other RNA viruses, which may be explained by the possession of nonstructural protein 14 (nsp14) which encodes a proofreading RNase called ExoN and is crucial for CoV RNA synthesis and maintaining CoV replication fidelity (31, 32). Positive selection, especially in the spike protein and RBD, may have been important during HCoV-229E evolution at the receptor-binding interphase, probably to adapt to new environmental changes or evade the immune system. This is in contrast to the belief that human CoV is relatively stable as it has been circulating in humans for decades. Previous HCoV-229E spike-receptor structural analysis focused only on the interaction of the RBD in complex with hAPN (21, 33), but the role of positively selected residues outside the RBD remains unclear. Further investigations are warranted to understand the possible effects of mutations of these two codons outside the RBD in spike-receptor binding and HCoV-229E evolution.

The present study revealed the estimation of divergence times for human- and animal-associated 229E viruses. Similarly to SARS-CoV, HCoV-229E may have also originated from bats. A previous study has reported CoVs closely related to HCoV-229E in bats of the genus *Hipposideros* in Africa (34, 35). These bat viruses shared >90% amino acid sequence identities in the seven conserved replicase domains for CoV species demarcation, suggesting that they belong to the same CoV species as HCoV-229E (35). Moreover, CoVs even closer to HCoV-229E have been identified in alpacas in the United States and dromedary camels in Africa and the Arabian Peninsula (15, 36). However, it remains to be determined if dromedaries or related animals have served as an intermediate host for bat-to-human transmission, or if both humans and dromedaries have acquired 229E-related CoVs independently from a common ancestor. Alpaca and dromedary camel viruses showed a similar deletion in the S1 domain of spike protein as HCoV-229E, which was not found in bat viruses. An additional putative ORF8 downstream of the nucleocapsid gene was present in bat and dromedary viruses but not in HCoV-229E. The only exception was a small in-frame deletion found in a dromedary strain, KCSP1, and a partial deletion in alpaca virus (15, 35). Although these features may suggest bat-to-camelid-to-human transmission, our molecular dating analysis showed a relatively recent time of divergence (tMRCA 1999) among dromedary/alpaca viruses. Therefore, it is possible that yet-unidentified animals may have served as the intermediate hosts for bat-to-human and bat-to-camelid transmission. This is also supported by the monophyletic clades formed by human and camelid viruses, respectively, in previous phylogenetic studies (15). Yet, one limitation of the molecular clock analysis is that dromedary/alpaca viruses available for analysis were detected only after 2008, which may affect the accuracy of tMRCA estimation. Moreover, frequent recombination among coronaviruses may pose problem for divergence time estimation analysis. Multiple recombination events occurred involving HCoV-229E, bat 229E-related CoVs, and alpaca 229E-related CoVs, with major recombination breakpoints occurring within ORF1ab and the beginning of the S gene (35). Phylogenetic analysis using specific nsp regions such as RdRp, 3CLpro, and helicase demonstrated that the topologies of nsp regions were incongruent because of multiple recombination events as opposed to that of ORF1ab (data not shown). This scenario was also observed in the work of Corman et al. (35), where a monophyletic clade was formed by human and alpaca 229E-related CoV in ORF1ab and S genes but alpaca 229E-related CoV was clustered with bat 229E-related CoVs in M, E, N, and ORF4 genes. Therefore, the diverse topologies and frequent recombination events among coronaviruses may affect the accuracy of tMRCA estimation.

Since human CoVs are seldom included in routine respiratory virus detection panels in clinical laboratories, the prevalence and importance of HCoV-229E may have been underestimated. The causative role of HCoV-229E in respiratory tract infections was first confirmed in the 1960s when healthy volunteers who received inoculations of cultured HCoV-229E developed symptoms of the common cold (37). While immunocompetent adults can be infected with HCoV-229E, immunocompromised patients as well as infants and the elderly are particularly susceptible, with higher chance of severe disease (38, 39). The prevalence of HCoV-229E varies widely among different countries and study periods, ranging from 4.6 to 17.0% among all coronaviruses detected in respiratory specimens (40–44). While other human coronaviruses are known to exhibit seasonal patterns, the seasonality of HCoV-229E has been less clearly understood, partly due to the low detection rate and limited epidemiological data. While a winter and early spring seasonality has been suggested, detection of HCoV-229E has been found to disperse in all four seasons in other studies (40, 42, 43). In the present study, the clinical spectrum of diseases of HCoV-229E infection is similar to that of HCoV-HKU1 and HCoV-OC43 (10, 25, 40, 41), with most patients in the extremes of age or with underlying diseases, although healthy, young patients were occasionally affected. In contrast to the traditional belief that human coronaviruses are mostly associated with mild URTI or common cold, a significant proportion of patients in the present study had severe infections such as pneumonia or complications such as exacerbation of COPD. While no particular genogroup of strains was associated with more severe infections, further studies with inclusion of more cases may allow better understanding of the pathogenicity of the different genogroups.

## MATERIALS AND METHODS

### Patient samples.

A nasopharyngeal swab from the first fatal COVID-19 case in Hong Kong and nasopharyngeal aspirates (NPAs) from 41 hospitalized patients with HCoV-229E infections from years 2004 to 2019 were included (40, 41). This study was approved by the Institutional Review Board of the University of Hong Kong/Hospital Authority Hong Kong West Cluster (UW 16-365) and the Research Ethics Committee of Hong Kong East Cluster (HKEC-2016-041).

### RNA extraction.

Viral RNA was extracted from the NPAs of the corresponding patients using the QIAamp viral RNA minikit (Qiagen, Hilden, Germany). The RNA pellet was eluted in 60 μl of DNase-free, RNase-free double-distilled water and was used as the template for RT-PCR.

### Genome sequencing of SARS-CoV-2 and HCoV-229E detected from the fatal case and phylogenetic analysis.

RNA was converted to cDNA by a combined random-priming and oligo(dT) priming strategy and specific primers for SARS-CoV-2 and HCoV-229E, respectively. The complete genomes of SARS-CoV-2 and HCoV-229E from the fatal COVID-19 case were amplified and sequenced using primers shown in Tables S1 and S2 in the supplemental material. Both forward and reverse strands of the PCR products were sequenced twice with an ABI Prism 3700 DNA analyzer (Applied Biosystems) using the respective PCR primers. Sequences were assembled and manually edited to produce the final sequences of the viral genomes using Geneious R11 (Biomatters, Auckland, New Zealand). A phylogenetic tree using nucleotide sequences was constructed using the maximum likelihood method in MEGA 7.0 with the GTR + G + I substitution model. Bootstrap replicates of 1,000 were selected for assessment of the robustness of branches.

10.1128/mSphere.00819-20.1 TABLE S1 Primers used for complete genome sequencing of HCoV-229E strain HK20-42. Download Table S1, DOCX file, 0.01 MB. Copyright © 2021 Lau et al. 2021 Lau et al. https://creativecommons.org/licenses/by/4.0/ This content is distributed under the terms of the Creative Commons Attribution 4.0 International license.

10.1128/mSphere.00819-20.2 TABLE S2 Primers used for complete genome sequencing of SARS-CoV-2 strain HK13. Download Table S2, DOCX file, 0.01 MB. Copyright © 2021 Lau et al. 2021 Lau et al. https://creativecommons.org/licenses/by/4.0/ This content is distributed under the terms of the Creative Commons Attribution 4.0 International license.

### RT-PCR and sequencing of complete RdRp, S, and N genes of HCoV-229E and phylogenetic analysis.

The RNA was converted to cDNA by a combined random-priming and oligo(dT) priming strategy. The complete RdRp, S, and N genes of HCoV-229E from 41 NPAs were amplified and sequenced using the primers shown in Table S3 and strategies described previously (10, 40, 41). The nucleotide and deduced amino acid sequences of the RdRp, S, and N genes were compared to those of HCoV-229E sequences available in GenBank. Phylogenetic trees using nucleotide sequences were constructed using the maximum likelihood method in MEGA 7.0 with TN93 + G model for RdRp, TN93 + G model for S, and T92 + G model for N genes. The substitution models were selected according to the Akaike information criterion (AIC) implemented in MEGA 7.0 (45). The robustness of branches was assessed by bootstrap analysis with 1,000 replicates.

10.1128/mSphere.00819-20.3 TABLE S3 Primers used for PCR and sequencing of the complete RdRp, S, and N genes of the 41 HCoV-229E strains. Download Table S3, DOCX file, 0.01 MB. Copyright © 2021 Lau et al. 2021 Lau et al. https://creativecommons.org/licenses/by/4.0/ This content is distributed under the terms of the Creative Commons Attribution 4.0 International license.

### Selective pressure analysis.

The number of synonymous substitutions per synonymous site, *K_s_*, and the number of nonsynonymous substitutions per nonsynonymous site, *K_a_*, for RdRp, S, and N genes were calculated using the Nei and Gojobori substitution model with Jukes-Cantor correction in MEGA 7.0 as described previously (46). Sites under positive selection were inferred using single-likelihood ancestor counting (SLAC), fixed-effects likelihood (FEL), and random-effects likelihood (REL) methods as implemented in the DataMonkey server (available online at https://www.datamonkey.org/). Positive selection for a site was considered to be statistically significant if the *P* value was <0.05 for SLAC and FEL methods and the Bayes factor was >50 for the REL method. The mixed-effects model of evolution (MEME) was used to detect positively selected sites under episodic diversifying selection in particular positions among different clades within a phylogenetic tree, even when positive selection was not evident across the entire tree. Positively selected sites with a *P* value of <0.05 were reported.

### Estimation of divergence time.

Divergence time was calculated using complete ORF1ab sequence data of bat 229E-related CoVs, camel 229E-related CoVs, alpaca-related CoV, and HCoV-229E, with the Bayesian Markov chain Monte Carlo (MCMC) approach as implemented in BEAST (version 1.8.2) as described previously (25, 47). Analyses were performed using the GTR + G model with coding sequences partitioned into the first plus second positions versus the third position, and rate variations between sites were described by a four-category discrete gamma distribution using the uncorrelated exponential relaxed molecular clock and exponential growth coalescent model. The MCMC run was 2 × 10^8^ steps in length, with sampling every 1,000 steps. Convergence was assessed on the basis of the effective sampling size after a 10% burn-in with Tracer software version 1.7.1 (available online at http://tree.bio.ed.ac.uk/software/tracer/). The mean time of the most recent common ancestor (tMRCA) and the highest posterior density regions at 95% (HPD) were calculated. The tree was summarized in a target tree by using the Tree Annotator program (version 1.8.2) included in the BEAST package by choosing the tree with the maximum sum of posterior probabilities (maximum clade credibility) after a 10% burn-in.

### Accession number(s).

The complete genome sequences of SARS-CoV-2 and HCoV-229E and the complete RdRp, S, and N nucleotide sequences of the 41 HCoV-229E strains were deposited in GenBank under accession no. MT786446 and MT797634 to MT797757.
