# Supplementary material for: Molecular Evolution of Human Coronavirus 229E in Hong Kong and a Fatal COVID-19 Case Involving Coinfection with a Novel Human Coronavirus 229E Genogroup
Source: mSphere. 2021 Feb 10;6(1):e00819-20. doi: 10.1128/mSphere.00819-20 (PMC8544887; doi:10.1128/mSphere.00819-20)
Supplement: TABLE S1 [file msphere.00819-20-st001.docx]

| **Primer** | **Primer sequence (5’ – 3’)** | **Primer direction** | **Gene** |
| --- | --- | --- | --- |
| LPW39015 | ACTTAAGTACCTTATCTATCTACAGATAGA | Forward | 5’ UTR |
| LPW27251 | CCAACCACCCATGAAGCAT | Reverse | nsp 1 |
| LPW38847 | TAGTCTAAGGGTTTCGTGTTCC | Forward | 5’ UTR |
| LPW38848 | GGAACCATTTGTCATAACAGGAG | Reverse | nsp 2 |
| LPW38849 | TGGTTCTGTTCTTGAAATGGC | Forward | nsp 2 |
| LPW38850 | TGTGGTGACAAGTTTTACAAGCG | Reverse | nsp 2 |
| LPW38851 | GATTGCTTTGTTGCTTCTTCC | Forward | nsp 2 |
| LPW38852 | CCCATTTGTCAACATAGTCTCTC | Reverse | nsp 2 |
| LPW38853 | GGCTACTTCCGTCTTATGG | Forward | nsp 2 |
| LPW38854 | CAAACTCAAGTCATTACCGCC | Reverse | nsp 3 |
| LPW38855 | AAAAGCCGCTGGTGGTAAAG | Forward | nsp 2–3 |
| LPW38856 | GGTAATGACCACAAGACACAG | Reverse | nsp 3 |
| LPW38857 | AAACCAGTCTGCTCATCAAT | Forward | nsp 3 |
| LPW38858 | AGCAGCACTAAGCACACC | Reverse | nsp 3 |
| LPW38859 | CTCTATGCTTACTTTGGATGACC | Forward | nsp 3 |
| LPW38860 | TGTCTTCAGCATCACCCTTGT | Reverse | nsp 3 |
| LPW39021 | AGGTCTTGATGCTGCGTG | Forward | nsp 3 |
| LPW38862 | CTTTGTGGTGCTTTGGC | Reverse | nsp 3 |
| LPW38863 | TAGACTTTTGAGTGGTGTTGC | Forward | nsp 3 |
| LPW38864 | TAGCACTCTTAGAACACGCAAT | Reverse | nsp 3 |
| LPW38865 | GCTTCTTGCTGTATTTTGTTGC | Forward | nsp 3 |
| LPW38866 | AGGTTTAGCATACGAACTGACA | Reverse | nsp 3 |
| LPW38867 | GTGAACTTTTGTCCACTTTGTC | Forward | nsp 3 |
| LPW38868 | ATTGTTACCACCCAAACCTTC | Reverse | nsp 4 |
| LPW38869 | GAGTCACAACACCTGAAAAGT | Forward | nsp 4 |
| LPW38980 | TAGGCATAACAAGCACAACGG | Reverse | nsp 4 |
| LPW38981 | CTTTCTTGCTATGTTGACAGGTTTG | Forward | nsp 4 |
| LPW38982 | CTGTGAAACCATTAGCCTGTGC | Reverse | nsp 5 |
| LPW38873 | ATTGAACTCGGAAGTGGTAG | Forward | nsp 5 |
| LPW38874 | AACACCATCAAAGTAAGACACG | Reverse | nsp 6 |
| LPW38875 | TGCTGGTTATGCTACTTTGTGC | Forward | nsp 6 |
| LPW38876 | GCACAAAACCATCTACCATCA | Reverse | nsp 8 |
| LPW38877 | GAGAACGACTCCATTTTGC | Forward | nsp 7 |
| LPW38878 | CTACCAGAACCATTAGTCAACATC | Reverse | nsp 10 |
| LPW38879 | CTGTTTTGGGTTACATTGGTGC | Forward | nsp 9 |
| LPW38880 | AAAGCAGCATACACACGG | Reverse | RdRp |
| LPW38881 | GCTCTGCGTAACTTTGATGA | Forward | RdRp |
| LPW38882 | AGCGACAGAAAAACAAACAGTG | Reverse | RdRp |
| LPW38883 | ACACACTCTTCGCAACCACAAT | Forward | RdRp |
| LPW18824 | TTTGAATAAACAACCTCGGTCAA | Reverse | RdRp |
| LPW19676 | GTTATGAGGAACAAGATGCTATTT | Forward | RdRp |
| LPW38886 | ATAGCCAGAGAAACATAGCG | Reverse | RdRp |
| LPW38887 | GCTAAGTGTTGGACTGAGGAAG | Forward | RdRp |
| LPW38888 | CAGTGGATTTGTAAGTAACCGT | Reverse | nsp 13 |
| LPW38889 | GGTGAGTTTGTGTTTGAGAAAGTAG | Forward | nsp 13 |
| LPW38890 | TCACTACCTTGAGCAGAATCC | Reverse | nsp 13 |
| LPW38891 | GGTAGTGTTCAGGTAGACAATGG | Forward | nsp 13 |
| LPW38892 | CCATTCCTAACCAACCTCTGAC | Reverse | nsp 14 |
| LPW38893 | GCCACTACTTATTTGTCATTGTCTG | Forward | nsp 14 |
| LPW21540 | ATTGTTCTCCAGGTGGTGC | Reverse | nsp 14 |
| LPW38984 | AGTGTTGTAAAACCTGTTCGTGC | Forward | nsp 14 |
| LPW38985 | TCATAGTAGAAAAACGGTGCTGG | Reverse | nsp 14 |
| LPW21539 | TAGATGTTTGGCTGTGTATGACT | Forward | nsp 14 |
| LPW38896 | CAGCACCAACAAATGAACC | Reverse | nsp 15 |
| LPW38897 | TTGGTGGTGCTGTATGTTC | Forward | nsp 14 |
| LPW38898 | AATCAACAAGTGTAAACCGCC | Reverse | nsp 15 |
| LPW38899 | TTTTTGCTAAGAGGAAGGTTGG | Forward | nsp 15 |
| LPW38900 | CACCAGTAACACTAAAATCAGC | Reverse | nsp 16 |
| LPW38901 | TACACTTGGGTGCTGGCTC | Forward | nsp 16 |
| LPW20312 | ATAAAACACAACAGCACCATAAG | Reverse | S |
| LPW19059 | CTTGTTAGGAGTGGTAAGTTGCT | Forward | nsp 16 |
| LPW38902 | AACTCACGAACTGTCTTAGGTAG | Reverse | S |
| LPW19061 | ACTACCTAAGACAGTTCGTGAG | Forward | S |
| LPW19158 | TAGCGAAAAACATACACTGCC | Reverse | S |
| LPW19157 | TGTTGACACATCACACTTCACTAC | Forward | S |
| LPW19160 | TAAATACTCAACCTGGACCGAA | Reverse | S |
| LPW38954 | TGTATCTGGTGTGGGTGTTATTCG | Forward | S |
| LPW38955 | TCAAGTCTGTCATAGATAGCCTG | Reverse | S |
| LPW38995 | CTGTTGTTGGTGCTATGTTGTCTG | Forward | S |
| LPW38998 | AAGCCCCTCAGGAGCAGC | Reverse | S |
| LPW38912 | GTGTCAAATCCCAGTCTAAGCG | Forward | S |
| LPW38913 | AGCAACCAGTAGAACAACAAC | Reverse | S |
| LPW38914 | CAGTCGTGCTCATCTTTGTGG | Forward | S |
| LPW38915 | ATAACGAGTGTTGATACGGC | Reverse | M |
| LPW38916 | GCACATAGACCCTTTCCC | Forward | E |
| LPW38917 | AATCCACCCGTTTGCCC | Reverse | N |
| LPW38918 | GCCCTTTGCTTGTTGATAGTGA | Forward | N |
| LPW38919 | ACTTCATCACGCACTGGTTC | Reverse | N |
| LPW19060 | CACATTGTTTCCAAAGAGTCAG | Forward | N |
| LPW00418 | GACCACGCGTATCGATGTCGACTTTTTTTTTTTTTTTTV | Reverse | 3' UTR |
