# Supplementary material for: Molecular Evolution of Human Coronavirus 229E in Hong Kong and a Fatal COVID-19 Case Involving Coinfection with a Novel Human Coronavirus 229E Genogroup
Source: mSphere. 2021 Feb 10;6(1):e00819-20. doi: 10.1128/mSphere.00819-20 (PMC8544887; doi:10.1128/mSphere.00819-20)
Supplement: TABLE S2 [file msphere.00819-20-st002.docx]

| **Primer** | **Primer sequence (5’ – 3’)** | **Primer direction** | **Gene** |
| --- | --- | --- | --- |
| LPW38722 | ATTAAAGGTTTATACCTTCCCAGGT | Forward | 5’ UTR |
| LPW38723 | CCAGCTCCTTTATTACCGTTCTTAC | Reverse | nsp 1 |
| LPW38724 | GGTCATGTTATGGTTGAGCTGGTAG | Forward | nsp 1 |
| LPW38725 | AAAATTTGGACATTCCCCATTGAAG | Reverse | nsp 2 |
| LPW38726 | TTGACACTAAGAGGGGTGTATACTG | Forward | nsp 2 |
| LPW38727 | GCACAGAATTTTGAGCAGTTTCAAG | Reverse | nsp 2 |
| LPW38728 | CTAAAAAAGGTGCCTGGAATATTGG | Forward | nsp 2 |
| LPW38729 | TTCTGTACCGAGTTCAACTGTATAG | Reverse | nsp 3 |
| LPW38730 | TTTGGTGATGACACTGTGATAGAAG | Forward | nsp 3 |
| LPW38731 | AGTAGACATTTGTGCGAACAGTATC | Reverse | nsp 3 |
| LPW38732 | AAGACATTCAACTTCTTAAGAGTGC | Forward | nsp 3 |
| LPW38733 | GCTTCTTCCAAATTTAAGCCATGTG | Reverse | nsp 3 |
| LPW38734 | GTGTGGTTGATTATGGTGCTAGATT | Forward | nsp 3 |
| LPW38735 | AACATCACCTAACTCACCTACTGTC | Reverse | nsp 3 |
| LPW38736 | TTGAAGTTTAATCCACCTGCTCTAC | Forward | nsp 3 |
| LPW38737 | TCCTCTGACTTCAGTACATCAAACG | Reverse | nsp 3 |
| LPW38738 | TAACAATGCAACTAATAAAGCCACG | Forward | nsp 3 |
| LPW38739 | GAATATATGCCAAAAACCACTCTGC | Reverse | nsp 3 |
| LPW38740 | CCTTGTAGTGTTTGTCTTAGTGGTT | Forward | nsp 3 |
| LPW38741 | ACACATTCTTTGCAAGTTCAGCTTC | Reverse | nsp 3 |
| LPW38742 | GCGGAAGTTGCAGTTAAAATGTTTG | Forward | nsp 3 |
| LPW38743 | AAATTGTACATTCAGCAGCCAAAAC | Reverse | nsp 4 |
| LPW38744 | GCACGATATTACGCACAACTAATGG | Forward | nsp 4 |
| LPW38745 | AGGTAATAGCACATCACTACGCAAC | Reverse | nsp 4 |
| LPW38746 | AAGAGACGTGTAGTCTTTAATGGTG | Forward | nsp 4 |
| LPW38747 | CCTAGTATGTCAACATGGTCTTGTG | Reverse | nsp 5 |
| LPW38748 | GGTTGTACGCTGCTGTTATAAATGG | Forward | nsp 5 |
| LPW38749 | GAGTCAGTCTAAAGTAGCGGTTGAG | Reverse | nsp 6 |
| LPW38750 | ATTGCCCTATTTTCTTCATAACTGG | Forward | nsp 6 |
| LPW38751 | TAAGTAAATGTTGTACCATCACACG | Reverse | nsp 8 |
| LPW38752 | CCTTGAACATAATACCTCTTACAAC | Forward | nsp 8 |
| LPW38753 | ATCACAACTACAGCCATAACCTTTC | Reverse | nsp 10 |
| LPW38790 | GTTGCCACATAGATCATCCAAATCC | Forward | nsp 10 |
| LPW38791 | ACAATTTGGGTGGTATGTCTGATCC | Reverse | RdRp |
| LPW38754 | TTAACCTTGACCAGGGCTTTAACTG | Forward | RdRp |
| LPW38755 | TACTACAGATAGAGACACCAGCTAC | Reverse | RdRp |
| LPW38756 | ATGAGTTATGAGGATCAAGATGCAC | Forward | RdRp |
| LPW38757 | GCATCTATAGCTAAAGACACGAACC | Reverse | RdRp |
| LPW38758 | TACCCAGATCCATCAAGAATCCTAG | Forward | RdRp |
| LPW38759 | CTCATCTGAGATATTGAGTGTTGGG | Reverse | nsp 13 |
| LPW38760 | ATGTTGGTGATTATTTTGTGCTGAC | Forward | nsp 13 |
| LPW38761 | CTGTTTACATTACAAGAGTGAGCTG | Reverse | nsp 13 |
| LPW38762 | CTCAAACTGTTGATTCATCACAGGG | Forward | nsp 13 |
| LPW38763 | CTAGACACCTAGTCATGATTGCATC | Reverse | nsp 14 |
| LPW38764 | GGGTTTTACAGGTAACCTACAAAGC | Forward | nsp 14 |
| LPW38765 | TTTCACCTCTGGTACTGGTTTAATG | Reverse | nsp 15 |
| LPW38766 | AGTTGATGGTGTTGATGTAGAATTG | Forward | nsp 15 |
| LPW38767 | TGTAAAGATTAGGCATAGCAACACC | Reverse | nsp 16 |
| LPW38768 | AGAAATTTCATTTATGCTTTGGTGT | Forward | nsp 16 |
| LPW38769 | GGGTAATTGAGTTCTGGTTGTAAGA | Reverse | nsp 16 |
| LPW38770 | GTTGTTATTTCTAGTGATGTTCTTG | Forward | nsp 16 |
| LPW38771 | GTTGGACTCTAAAGTTAGAAGTTTG | Reverse | nsp 16 |
| LPW38772 | GAAAATGGAACCATTACAGATGCTG | Forward | S |
| LPW38773 | AACATCCTGATAAAGAACAGCAACC | Reverse | S |
| LPW38774 | AGAGACATTGCTGACACTACTGATG | Forward | S |
| LPW38775 | CAGAAGTGATTGTACCCGCTAACAG | Reverse | S |
| LPW38776 | CTTGGTGATATTGCTGCTAGAGACC | Forward | S |
| LPW38777 | TAATGCCAGAGATGTCACCTAAATC | Reverse | S |
| LPW38778 | TGATCCTTTGCAACCTGAATTAGAC | Forward | S–Orf3a |
| LPW38779 | CAGCTGGTAATAGTCTGAAGTGAAG | Reverse | Orf3a |
| LPW38780 | GTGATGGCACAACAAGTCCTATTTC | Forward | Orf3a |
| LPW38781 | TTACGAGTTCACTTTCTAGAAGCGG | Reverse | M |
| LPW38782 | TCATTGCTTCTTTCAGACTGTTTGC | Forward | M |
| LPW38783 | CAAGTGAGAACCAAAAGATAATAAG | Reverse | Orf7b |
| LPW38784 | TGCTTCACACTCAAAAGAAAGACAG | Forward | Orf7a |
| LPW38785 | TGTGATCTTTTGGTGTATTCAAGGC | Reverse | N |
| LPW38786 | ATGAAAGATCTCAGTCCAAGATGGT | Forward | N |
| LPW38787 | CTTGTGTGGTCTGCATGAGTTTAGG | Reverse | N |
| LPW38788 | CTTACCGCAGAGACAGAAGAAACAG | Forward | N |
| LPW38789 | TTTTTTTTTTTTTTTTTTGTCATTC | Reverse | 3’ UTR |
