# Supplementary material for: Molecular Evolution of Human Coronavirus 229E in Hong Kong and a Fatal COVID-19 Case Involving Coinfection with a Novel Human Coronavirus 229E Genogroup
Source: mSphere. 2021 Feb 10;6(1):e00819-20. doi: 10.1128/mSphere.00819-20 (PMC8544887; doi:10.1128/mSphere.00819-20)
Supplement: TABLE S3 [file msphere.00819-20-st003.docx]

| **Primer** | **Primer sequence (5’ – 3’)** | **Primer direction** | **Gene** |
| --- | --- | --- | --- |
| **RdRp gene** |  |  |  |
| LPW03209 | GTKTGTAWDGTYTGTGGTTGTT | Forward | nsp 10 |
| LPW19468 | CGGTGTATGTCCTCATTTTCTA | Reverse | RdRp |
| LPW18823 | TACACCATGATGGATTTGTGCTT | Forward | RdRp |
| LPW19469 | CGAAAAACCCCTGAGAACG | Reverse | RdRp |
| LPW19675 | CCTGCCTTGGTGGATAAAC | Forward | RdRp |
| LPW19676 | GTTATGAGGAACAAGATGCTATTT | Forward | RdRp |
| LPW18824 | TTTGAATAAACAACCTCGGTCAA | Reverse | RdRp |
| LPW02905 | GTGTGATAGAGCTATGCCCTCA | Forward | RdRp |
| LPW02906 | GTAACCAAGTCCAGCATAAGTT | Reverse | RdRp |
| LPW06092 | AGCATTTTTCTATGATGAT | Forward | RdRp |
| LPW03482 | CCNAHNACRTGRTCRTADGCRCAYTT | Reverse | nsp 13 |
|  |  |  |  |
| **S gene** |  |  |  |
| LPW19059 | CTTGTTAGGAGTGGTAAGTTGCT | Forward | nsp 16 |
| LPW20311 | CTTATGGTGCTGTTGTGTTTTAT | Forward | S |
| LPW20312 | ATAAAACACAACAGCACCATAAG | Reverse | S |
| LPW19061 | ACTACCTAAGACAGTTCGTGAG | Forward | S |
| LPW19062 | ATAAAAATGTCCTGTGCGT | Reverse | S |
| LPW19157 | TGTTGACACATCACACTTCACTAC | Forward | S |
| LPW19158 | TAGCGAAAAACATACACTGCC | Reverse | S |
| LPW20478 | CAAGTTTGATAGGTGGAGTGCT | Forward | S |
| LPW20652 | CTGTTGTTGGTGCTATGTTGTC | Forward | S |
| LPW20653 | GTATTGAAGCGGCTGATGTAA | Reverse | S |
| LPW19161 | GCTGATGCTGAACGAATGG | Forward | S |
| LPW19162 | TACTATGTTGGTCATTGCTTTGTT | Reverse | S |
| LPW20313 | TATGGCTTCTGTGGAAATGG | Forward | S |
| LPW20314 | CCACGCTTCAACATCCTTAT | Reverse | S |
| LPW20479 | TTCGCCTTGATAAGAGATTGAT | Reverse | 4a |
|  |  |  |  |
| **N gene** |  |  |  |
| LPW18510 | AGTGAGCTCTCCCATGAGCAA | Forward | M |
| LPW19608 | CAGGTGAAGTTTGAGATGGGT | Reverse | N |
| LPW19065 | TACAGGTTACGGTGTTAGGCG | Forward | N |
| LPW19060 | CACATTGTTTCCAAAGAGTCAG | Forward | N |
| LPW02197 | GCTCTTCCATTRTWGGCTCGTC | Reverse | 3’ UTR |
